# Effects of changes in diagnosis and registration on time trends in recorded childhood cancer incidence in Great Britain

**DOI:** 10.1038/bjc.2012.296

**Published:** 2012-08-16

**Authors:** M E Kroll, L M Carpenter, M F G Murphy, C A Stiller

**Affiliations:** 1 Childhood Cancer Research Group, Department of Paediatrics, University of Oxford, Oxford OX3 7LG, UK; 2 Cancer Epidemiology Unit, Nuffield Department of Medicine, University of Oxford, Oxford OX3 7LF, UK; 3 Department of Public Health, University of Oxford, Rosemary Rue Building, Oxford OX3 7LF, UK; 4 Nuffield College, University of Oxford, New Road, Oxford OX1 1NF, UK

**Keywords:** childhood cancer, childhood leukaemia, incidence, time trends, delayed diagnosis, cancer registration

## Abstract

**Background:**

Increases in recorded childhood cancer incidence are widely reported, but do not necessarily represent real increases in risk. Time trends might conceal underlying steps caused by changes in diagnosis and registration procedures.

**Methods:**

Using records from the National Registry of Childhood Tumours 1966–2005 (*N*=54 650), the age-sex-standardised rate for residents of Great Britain aged under 15 years was calculated by individual year of diagnosis for each cancer subtype, and the average annual percentage change (trend) was assessed. The timing of assumed step changes in rate was estimated by iterative Poisson regression, and compared graphically with the approximate timing of innovations previously identified from published sources.

**Results:**

Estimated timing of underlying steps approximately coincided with the following relevant innovations: biochemical assays, mid-1980s (hepatic and germ-cell cancer); diagnostic imaging, mid-1980s to early 1990s (intracranial/intraspinal tumours, neuroblastoma, soft-tissue sarcoma); revised cancer registration scheme, 1971 (leukaemia, bone and soft-tissue sarcoma); mandatory registration, 1993 (intracranial/intraspinal tumours, retinoblastoma, melanoma/carcinoma); cancer registration improvements, 2001 (leukaemia, renal and hepatic cancer).

**Conclusion:**

While the possibility of some real change in risk cannot be excluded, for many cancer subtypes the estimated timing of underlying step changes in rate appeared to correspond with changes in diagnosis or registration procedures. Childhood cancer may have been considerably under-recorded in the past.

Increases in recorded childhood cancer incidence are widely reported in the developed world. Exposure to some hypothesised risk factors, such as electromagnetic fields or unusual patterns of infection, might have become more prevalent over time. Alternatively, the increases might reflect changes in diagnosis and registration procedures, rather than true changes in cancer risk. The incidence trends have been attributed to mainly real increase in risk (dos Santos Silva *et al*, 1999; McNally *et al*, 2001a, 2001b; Steliarova-Foucher *et al*, 2004; Kaatsch *et al*, 2006; Kroll *et al*, 2006; Shah and Coleman, 2007; Spix *et al*, 2008), and also to improvement in case ascertainment (Linet *et al*, 1999; Adamson *et al*, 2005). In the United States, increases for childhood malignant brain tumours followed the introduction of magnetic resonance imaging during the mid-1980s (Smith *et al*, 1998).

Using national registry data from Great Britain, we estimated the timing of assumed underlying step changes in rate, and compared this with relevant innovations in diagnosis and registration procedure.

## Materials and methods

### Innovations in methods for diagnosis of childhood cancer

Standard paediatric oncology textbooks represented clinical practice in Great Britain (Marsden and Steward, 1968; Bloom *et al*, 1975; Voûte *et al*, 1986; Plowman and Pinkerton, 1992; Pinkerton *et al*, 2004). Nine important diagnostic innovations were identified (with year of first mention): urinary catecholamine assay for neuroblastoma (1975); alpha-fetoprotein assay for hepatic and germ-cell cancer, beta-human-chorionic-gonadotrophic-hormone assay for germ-cell cancer, computed tomography for intracranial and other solid tumours, immunohistochemistry for sub-classification of leukaemia, and ultrasound imaging for abdominal and other extra-cranial solid tumours (1986); immunohistochemistry for sub-classification of solid cancers, magnetic resonance imaging for intracranial/intraspinal and other solid tumours, and meta-iodobenzylguanidine scanning for neuroblastoma (1992).

### Innovations in registration procedures

The National Registry of Childhood Tumours (NRCT) records cancer diagnosed since 1962 in residents of Great Britain aged under 15 years (Stiller, 2007). Case notifications are received from defined multiple sources, including death certificates and specialist tumour registries, with careful matching and validation (Kroll *et al*, 2011). Principal sources during the study period were the regional and national all-ages (general) cancer registries, and the register of children seen by clinicians affiliated to the United Kingdom Children’s Cancer Study Group (UKCCSG), the organisation that coordinated paediatric oncology in the UK during 1977–2006.

Four changes in general cancer registration procedure were identified (ISD Scotland, 1998; Quinn *et al*, 2001; Office for National Statistics, 2010). A revised registration scheme was introduced on 1 January 1971 (i.e., for patients diagnosed from this date); responsibility for registration was transferred from region of treatment to region of residence on 1 January 1978; registration of National Health Service cancer patients became mandatory on 1 January 1993; the Department of Health published an action plan to promote improvements in the effectiveness of the system in 2001. The first full year of routine UKCCSG notification to the NRCT was 1978 (UKCCSG, 2002).

### Definition and classification of cases

Cases diagnosed from 1 January 1966 to 31 December 2005 were extracted from the NRCT, and grouped according to Level 1 of the International Classification of Childhood Cancer (Steliarova-Foucher *et al*, 2005) (Supplementary Table S1), excluding skin carcinoma and non-CNS disease treated as uncertain or benign in earlier classifications. Groups III and Xa were combined to include all intracranial/intraspinal neoplasms (CNS tumours). A subtotal group (non-CNS solid cancer) included all cases except leukaemia and CNS tumours.

### Statistical analysis

Annual rates were directly standardised by sex and age group (<1, 1–4, 5–9, 10–14 years) to a uniform population, and the average annual percentage change was estimated by Poisson regression. Assuming that underlying changes in rate occurred as a series of steps, the timing of the steps was estimated by the following iterative process. A set of time periods was defined, consisting initially of each single year of diagnosis. A categorical age-sex-adjusted Poisson model was fitted, allowing the rate to vary between time periods. The rates in each adjacent pair of periods were tested for inequality, and the pair with the highest *P*-value was amalgamated. Fitting and amalgamation were repeated until the *P*-value for inequality was below 0.01 in every pair of periods. At the end of this process the boundaries of the periods indicated the positions of a series of steps, which were graphically compared with the timing of relevant changes in techniques for diagnosis and registration.

## Results

In total, there were 54 650 eligible registrations (Supplementary Table S1). The proportion of ‘other/unspecified’ cancer (Group XII) was very small (0.5%). The age-sex-standardised overall annual rate increased by 1.0% per year during 1966–2005 (estimated 95% confidence interval 0.9–1.1), and was 8.4% higher in 2001–2005 (145.1 per million) than in 1996–2000 (133.9 per million). The step model indicated increases in 1972, 1976, 1987, 1993, and 2002 (Supplementary Figure S1).

**Figure 1:**
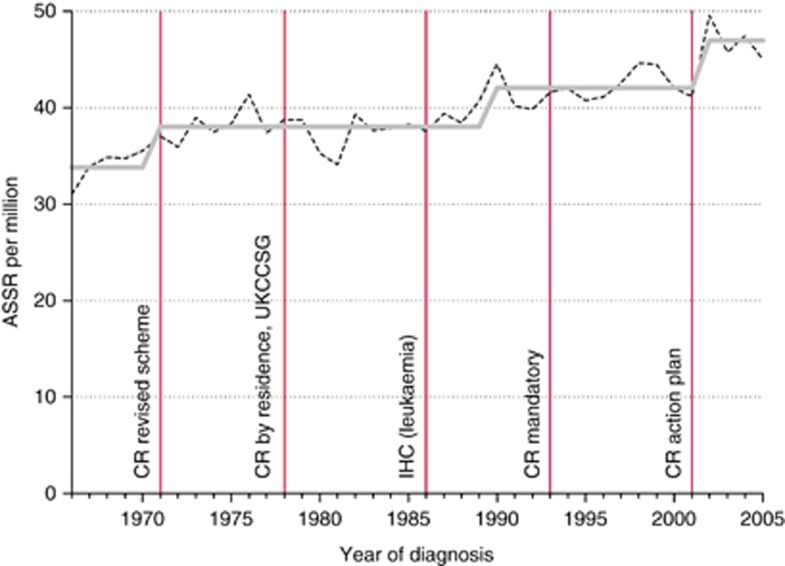
Recorded incidence of leukaemia under age 15 years, Great Britain 1966–2005. Age-sex-standardised rate (ASSR) by year of diagnosis: actual (dashed), step model (solid) line. Abbreviations: CR action plan, action plan for improvements in regional general cancer registration scheme; CR by residence, general cancer registration by region of residence, not of treatment; CR mandatory, general cancer registration becomes mandatory; CR revised scheme, revision of the regional general cancer registration scheme; IHC (leukaemia), immunohistochemistry for sub-classification of leukaemia; UKCCSG, ascertainment from UK Childrens’ Cancer Study Group patients’ register.

**Figure 2:**
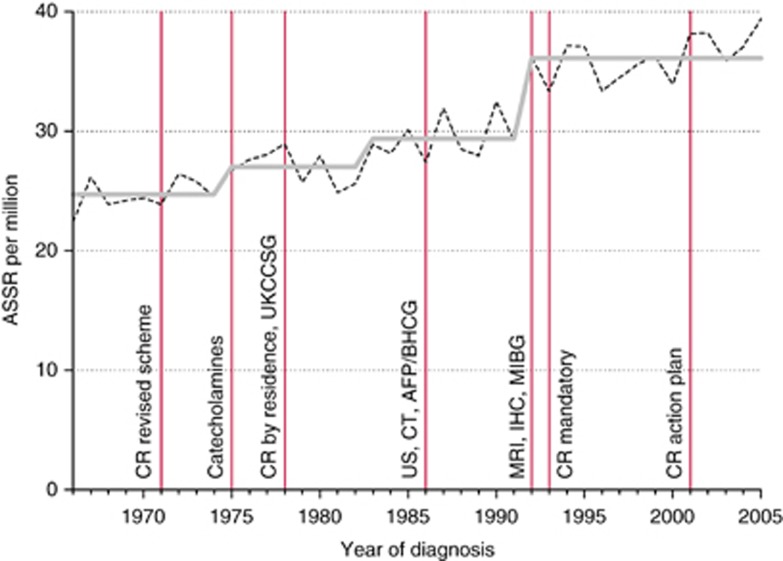
Recorded incidence of CNS tumours under age 15 years, Great Britain 1966–2005. Age-sex-standardised rate (ASSR) by year of diagnosis: actual (dashed), step model (solid) line. Abbreviations: AFP, alpha-fetoprotein assay for hepatic and germ-cell cancer; BHCG, beta human chorionic gonadotrophic hormone assay for germ-cell cancer; Catecholamines, urinary catecholamine assay for neuroblastoma; CR action plan, action plan for improvements in regional general cancer registration scheme; CR by residence, general cancer registration by region of residence, not of treatment; CR mandatory, general cancer registration becomes mandatory; CR revised scheme, revision of the regional general cancer registration scheme; CT, computed tomography for solid tumours; IHC, immunohistochemistry for sub-classification of solid cancer; MIBG, meta-iodobenzylguanidine scanning for neuroblastoma; MRI, magnetic resonance imaging for solid tumours; UKCCSG, ascertainment from UK Childrens’ Cancer Study Group patients’ register; US, ultrasound imaging for solid tumours.

**Figure 3:**
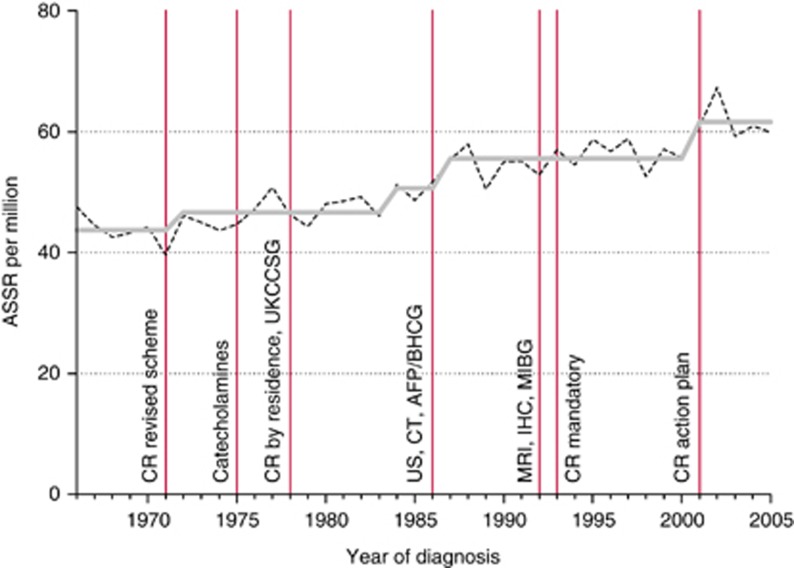
Recorded incidence of non-CNS solid cancer under age 15 years, Great Britain 1966–2005. Age-sex-standardised rate (ASSR) by year of diagnosis: actual (dashed), step model (solid) line. Abbreviations: AFP, alpha-fetoprotein assay for hepatic and germ-cell cancer; BHCG, beta human chorionic gonadotrophic hormone assay for germ-cell cancer; Catecholamines, urinary catecholamine assay for neuroblastoma; CR action plan, action plan for improvements in regional general cancer registration scheme; CR by residence, general cancer registration by region of residence, not of treatment; CR mandatory, general cancer registration becomes mandatory; CR revised scheme, revision of the regional general cancer registration scheme; CT, computed tomography for solid tumours; IHC, immunohistochemistry for sub-classification of solid cancer; MIBG, meta-iodobenzylguanidine scanning for neuroblastoma; MRI, magnetic resonance imaging for solid tumours; UKCCSG, ascertainment from UK Childrens’ Cancer Study Group patients’ register; US, ultrasound imaging for solid tumours.

For leukaemia, rates increased by 0.7% per year, with step model increases in 1971, 1990, and 2002 (Figure 1). For CNS tumours, rates increased by 1.3% per year, with relatively small increases in 1975 and 1983, followed by a larger step in 1992 (Figure 2). For non-CNS solid cancer, rates increased by 1.0% per year, with steps at 1972, 1984, 1987, and 2001 (Figure 3).

Rate increases occurred in all subtypes of non-CNS solid cancer, ranging from 0.5% per year for bone cancer to 2.5% for hepatic cancer (Supplementary Table S1). Timing and number of increases indicated by the step models varied between subtypes. For lymphoma there were two, in 1976 and 1997 (Supplementary Figure S2), whereas for neuroblastoma there was only one, in 1987 (Supplementary Figure S3). Equivalent results (Supplementary Figures S4–S10) were: retinoblastoma, 1993; renal cancer, 2000; hepatic cancer, 1984 and 2001; bone cancer 1972; soft-tissue sarcoma, 1972 and 1984; germ-cell/gonadal cancer, 1981; melanoma/carcinoma, 1975 and 1992.

## Discussion

### Overall trends

Recorded incidence of childhood cancer in Great Britain increased by 1% per year between 1966 and 2005, and was 8.4% greater in 2001–2005 than in 1996–2000. This suggests continuity of the increase previously reported by the NRCT for 1966–2000 (Stiller, 2007) and concurs with results from elsewhere, including the ACCIS consortium in Europe (Kaatsch *et al*, 2006) and the SEER registries in the United States (Ries *et al*, 2006). Step changes in the overall rate appeared to correspond with changes in diagnosis and registration procedures.

### Innovations in methods for diagnosis of childhood cancer

For solid tumours, several step changes approximately coincided with relevant diagnostic innovations: CNS tumours in the early 1980s (computed tomography) and early 1990s (magnetic resonance imaging, consistent with findings from the United States (Smith *et al*, 1998)), and non-CNS solid cancer in the mid-1980s (ultrasound and computed tomography). A notable increase in neuroblastoma rates around 1987 may in part represent cases that would previously have regressed spontaneously without diagnosis. An increase in soft-tissue sarcoma around 1984 may represent diagnosis by imaging at an earlier stage of disease, as this subtype is relatively common in adolescence. Increases in hepatic and germ-cell/gonadal cancer rates in the early to mid-1980s may reflect the introduction of relevant biochemical assays, and the UKCCSG study that (from 1979) pioneered their use for paediatric germ-cell malignancies in the UK (Mann *et al*, 1989). Conversely, bone cancer, which is usually detected by plain X-ray imaging (a procedure that hardly changed during the study period), incurred only a small increase over time, with no steps during 1973–2001.

For leukaemia, diagnostic change is less easy to define. The timing of the steps is probably influenced by a previously reported peak in annual rates in 1990 (Kroll *et al*, 2006). The only identified diagnostic innovation was immunohistochemistry, a technique used to identify subtypes of leukaemia rather than to make the initial diagnosis. However, there may have been a gradually increasing awareness of leukaemia as a cause of serious infection in childhood. In the past, some children with leukaemia might have died from infection without their leukaemia ever being diagnosed, and this proportion might have decreased as management of infection improved over time. Clinical evidence for under-diagnosis of acute lymphoblastic leukaemia in children from relatively deprived communities during the 1980s and 1990s is consistent with this hypothesis (Kroll *et al*, 2012).

### Innovations in registration procedures

Various step changes approximately coincided with improvements to the general cancer registration system: non-CNS solid cancer, bone and soft-tissue sarcoma and leukaemia around 1971 (the revised scheme); retinoblastoma, melanoma/carcinoma and CNS tumours around 1993 (mandatory registration); and non-CNS solid cancer, renal and hepatic cancer and leukaemia around 2001 (the action plan). Mandatory registration may have improved the ascertainment of children treated by clinicians not specialising in oncology: for example, general neurosurgeons for CNS tumours, ophthalmologists for retinoblastoma (outside the main centres), or dermatologists, endocrinologists and oral surgeons for melanoma/carcinoma. Increasing use of electronic pathology or haematology records may have contributed to the apparent rate increases. There was no evidence of any effect of changes in regional responsibilities, or the introduction of the UKCCSG register.

The rate increases in several cancer subtypes around 2001 are not fully understood. Case ascertainment for the mid-1990s appears to have been good, on the basis of a comparison with the UK Childhood Cancer Study (UKCCS), a case–control study that used slightly different sources. For the 2 years during which the UKCCS aimed for national coverage of childhood cancer in Great Britain (1993–1994), the NRCT registered 2955 cases (Groups I–XII), of which 739 were acute lymphoblastic leukaemia, whereas the UKCCS accrued 2650 cases, of which 722 were acute lymphoblastic leukaemia (Smith *et al* 2006). Capture–recapture estimates suggest that NRCT ascertainment of diagnosed cases was virtually complete by the mid-2000s (Kroll *et al*, 2011).

### Strengths and limitations

This 40-year study uses records from the national specialist childhood cancer registry for Great Britain. Step changes in rate were estimated using an appropriate objective statistical technique, and timing of relevant innovations was identified independently. The step model is plausible for modifications to the cancer registration system concerning cases diagnosed from a specific date. It is a reasonable approximation for some diagnostic innovations, particularly those not requiring costly equipment, but may be less appropriate for techniques that were introduced more gradually over time. Although innovations in diagnosis and registration methods are plausible explanations for most of the changes in recorded incidence, the possibility of some real increases should not be ruled out.

### Conclusions

While the possibility of some real change in risk cannot be excluded, for many cancer subtypes the estimated timing of underlying step changes in rate appeared to correspond with changes in diagnosis or registration procedures. Childhood cancer may have been considerably under-recorded in the past.
