# Supplementary material for: Effects of changes in diagnosis and registration on time trends in recorded childhood cancer incidence in Great Britain
Source: Br J Cancer. 2012 Aug 16;107(7):1159–62. doi: 10.1038/bjc.2012.296 (PMC3461151; doi:10.1038/bjc.2012.296)
Supplement: Supplementary Figures [file bjc2012296x1.doc]

**Figure S1. Total childhood cancer**. Recorded incidence under age 15 years, Great Britain 1966-2005. Age-sex-standardised rate (ASSR) by year of diagnosis: actual (dashed), step model (solid) line.

*Abbreviations*

AFP, Alpha-fetoprotein assay for hepatic and germ-cell cancer; BHCG, Βeta human chorionic gonadotrophic hormone assay for germ-cell cancer; Catecholamines, Urinary catecholamine assay for neuroblastoma; CR action plan, Action plan for improvements in regional general cancer registration scheme; CR by residence, General cancer registration by region of residence, not of treatment; CR mandatory, General cancer registration becomes mandatory; CR revised scheme, Revision of the regional general cancer registration scheme; CT, Computed tomography for solid tumours; IHC, Immunohistochemistry for sub-classification of solid cancer; MIBG, Meta-iodobenzylguanidine scanning for neuroblastoma; MRI, Magnetic resonance imaging for solid tumours; UKCCSG, Ascertainment from UK Childrens’ Cancer Study Group patients’ register; US, Ultrasound imaging for solid tumours.

**Figure S2. Lymphoma.** Recorded incidence under age 15 years, Great Britain 1966-2005.

Age-sex-standardised rate (ASSR) by year of diagnosis: actual (dashed), step model (solid) line.

*Abbreviations*

AFP, Alpha-fetoprotein assay for hepatic and germ-cell cancer; BHCG, Βeta human chorionic gonadotrophic hormone assay for germ-cell cancer; Catecholamines, Urinary catecholamine assay for neuroblastoma; CR action plan, Action plan for improvements in regional general cancer registration scheme; CR by residence, General cancer registration by region of residence, not of treatment; CR mandatory, General cancer registration becomes mandatory; CR revised scheme, Revision of the regional general cancer registration scheme; CT, Computed tomography for solid tumours; IHC, Immunohistochemistry for sub-classification of solid cancer; MIBG, Meta-iodobenzylguanidine scanning for neuroblastoma; MRI, Magnetic resonance imaging for solid tumours; UKCCSG, Ascertainment from UK Childrens’ Cancer Study Group patients’ register; US, Ultrasound imaging for solid tumours.

**Figure S3. Neuroblastoma.** Recorded incidence under age 15 years, Great Britain 1966-2005. Age-sex-standardised rate (ASSR) by year of diagnosis: actual (dashed), step model (solid) line.

*Abbreviations*

AFP, Alpha-fetoprotein assay for hepatic and germ-cell cancer; BHCG, Βeta human chorionic gonadotrophic hormone assay for germ-cell cancer; Catecholamines, Urinary catecholamine assay for neuroblastoma; CR action plan, Action plan for improvements in regional general cancer registration scheme; CR by residence, General cancer registration by region of residence, not of treatment; CR mandatory, General cancer registration becomes mandatory; CR revised scheme, Revision of the regional general cancer registration scheme; CT, Computed tomography for solid tumours; IHC, Immunohistochemistry for sub-classification of solid cancer; MIBG, Meta-iodobenzylguanidine scanning for neuroblastoma; MRI, Magnetic resonance imaging for solid tumours; UKCCSG, Ascertainment from UK Childrens’ Cancer Study Group patients’ register; US, Ultrasound imaging for solid tumours.

**Figure S4. Retinoblastoma.** Recorded incidence under age 15 years, Great Britain 1966-2005. Age-sex-standardised rate (ASSR) by year of diagnosis: actual (dashed), step model (solid) line.

*Abbreviations*

AFP, Alpha-fetoprotein assay for hepatic and germ-cell cancer; BHCG, Βeta human chorionic gonadotrophic hormone assay for germ-cell cancer; Catecholamines, Urinary catecholamine assay for neuroblastoma; CR action plan, Action plan for improvements in regional general cancer registration scheme; CR by residence, General cancer registration by region of residence, not of treatment; CR mandatory, General cancer registration becomes mandatory; CR revised scheme, Revision of the regional general cancer registration scheme; CT, Computed tomography for solid tumours; IHC, Immunohistochemistry for sub-classification of solid cancer; MIBG, Meta-iodobenzylguanidine scanning for neuroblastoma; MRI, Magnetic resonance imaging for solid tumours; UKCCSG, Ascertainment from UK Childrens’ Cancer Study Group patients’ register; US, Ultrasound imaging for solid tumours.

**Figure S5. Renal cancer.** Recorded incidence under age 15 years, Great Britain 1966-2005. Age-sex-standardised rate (ASSR) by year of diagnosis: actual (dashed), step model (solid) line.

*Abbreviations*

AFP, Alpha-fetoprotein assay for hepatic and germ-cell cancer; BHCG, Βeta human chorionic gonadotrophic hormone assay for germ-cell cancer; Catecholamines, Urinary catecholamine assay for neuroblastoma; CR action plan, Action plan for improvements in regional general cancer registration scheme; CR by residence, General cancer registration by region of residence, not of treatment; CR mandatory, General cancer registration becomes mandatory; CR revised scheme, Revision of the regional general cancer registration scheme; CT, Computed tomography for solid tumours; IHC, Immunohistochemistry for sub-classification of solid cancer; MIBG, Meta-iodobenzylguanidine scanning for neuroblastoma; MRI, Magnetic resonance imaging for solid tumours; UKCCSG, Ascertainment from UK Childrens’ Cancer Study Group patients’ register; US, Ultrasound imaging for solid tumours.

**Figure S6. Hepatic cancer.** Recorded incidence under age 15 years, Great Britain 1966-2005. Age-sex-standardised rate (ASSR) by year of diagnosis: actual (dashed), step model (solid) line.

*Abbreviations*

AFP, Alpha-fetoprotein assay for hepatic and germ-cell cancer; BHCG, Βeta human chorionic gonadotrophic hormone assay for germ-cell cancer; Catecholamines, Urinary catecholamine assay for neuroblastoma; CR action plan, Action plan for improvements in regional general cancer registration scheme; CR by residence, General cancer registration by region of residence, not of treatment; CR mandatory, General cancer registration becomes mandatory; CR revised scheme, Revision of the regional general cancer registration scheme; CT, Computed tomography for solid tumours; IHC, Immunohistochemistry for sub-classification of solid cancer; MIBG, Meta-iodobenzylguanidine scanning for neuroblastoma; MRI, Magnetic resonance imaging for solid tumours; UKCCSG, Ascertainment from UK Childrens’ Cancer Study Group patients’ register; US, Ultrasound imaging for solid tumours.

**Figure S7. Bone cancer.** Recorded incidence under age 15 years, Great Britain 1966-2005. Age-sex-standardised rate (ASSR) by year of diagnosis: actual (dashed), step model (solid) line.

*Abbreviations*

AFP, Alpha-fetoprotein assay for hepatic and germ-cell cancer; BHCG, Βeta human chorionic gonadotrophic hormone assay for germ-cell cancer; Catecholamines, Urinary catecholamine assay for neuroblastoma; CR action plan, Action plan for improvements in regional general cancer registration scheme; CR by residence, General cancer registration by region of residence, not of treatment; CR mandatory, General cancer registration becomes mandatory; CR revised scheme, Revision of the regional general cancer registration scheme; CT, Computed tomography for solid tumours; IHC, Immunohistochemistry for sub-classification of solid cancer; MIBG, Meta-iodobenzylguanidine scanning for neuroblastoma; MRI, Magnetic resonance imaging for solid tumours; UKCCSG, Ascertainment from UK Childrens’ Cancer Study Group patients’ register; US, Ultrasound imaging for solid tumours.

**Figure S8. Soft-tissue sarcoma.** Recorded incidence under age 15 years, Great Britain 1966-2005. Age-sex-standardised rate (ASSR) by year of diagnosis: actual (dashed), step model (solid) line.

*Abbreviations*

AFP, Alpha-fetoprotein assay for hepatic and germ-cell cancer; BHCG, Βeta human chorionic gonadotrophic hormone assay for germ-cell cancer; Catecholamines, Urinary catecholamine assay for neuroblastoma; CR action plan, Action plan for improvements in regional general cancer registration scheme; CR by residence, General cancer registration by region of residence, not of treatment; CR mandatory, General cancer registration becomes mandatory; CR revised scheme, Revision of the regional general cancer registration scheme; CT, Computed tomography for solid tumours; IHC, Immunohistochemistry for sub-classification of solid cancer; MIBG, Meta-iodobenzylguanidine scanning for neuroblastoma; MRI, Magnetic resonance imaging for solid tumours; UKCCSG, Ascertainment from UK Childrens’ Cancer Study Group patients’ register; US, Ultrasound imaging for solid tumours.

**Figure S9. Gonadal/germ-cell cancer.** Recorded incidence under age 15 years, Great Britain 1966-2005. Age-sex-standardised rate (ASSR) by year of diagnosis: actual (dashed), step model (solid) line.

*Abbreviations*

AFP, Alpha-fetoprotein assay for hepatic and germ-cell cancer; BHCG, Βeta human chorionic gonadotrophic hormone assay for germ-cell cancer; Catecholamines, Urinary catecholamine assay for neuroblastoma; CR action plan, Action plan for improvements in regional general cancer registration scheme; CR by residence, General cancer registration by region of residence, not of treatment; CR mandatory, General cancer registration becomes mandatory; CR revised scheme, Revision of the regional general cancer registration scheme; CT, Computed tomography for solid tumours; IHC, Immunohistochemistry for sub-classification of solid cancer; MIBG, Meta-iodobenzylguanidine scanning for neuroblastoma; MRI, Magnetic resonance imaging for solid tumours; UKCCSG, Ascertainment from UK Childrens’ Cancer Study Group patients’ register; US, Ultrasound imaging for solid tumours.

**Figure S10. Melanoma/carcinoma.** Recorded incidence under age 15 years, Great Britain 1966-2005. Age-sex-standardised rate (ASSR) by year of diagnosis: actual (dashed), step model (solid) line.

*Abbreviations*

AFP, Alpha-fetoprotein assay for hepatic and germ-cell cancer; BHCG, Βeta human chorionic gonadotrophic hormone assay for germ-cell cancer; Catecholamines, Urinary catecholamine assay for neuroblastoma; CR action plan, Action plan for improvements in regional general cancer registration scheme; CR by residence, General cancer registration by region of residence, not of treatment; CR mandatory, General cancer registration becomes mandatory; CR revised scheme, Revision of the regional general cancer registration scheme; CT, Computed tomography for solid tumours; IHC, Immunohistochemistry for sub-classification of solid cancer; MIBG, Meta-iodobenzylguanidine scanning for neuroblastoma; MRI, Magnetic resonance imaging for solid tumours; UKCCSG, Ascertainment from UK Childrens’ Cancer Study Group patients’ register; US, Ultrasound imaging for solid tumours.
