# Supplementary material for: Effects of changes in diagnosis and registration on time trends in recorded childhood cancer incidence in Great Britain
Source: Br J Cancer. 2012 Aug 16;107(7):1159–62. doi: 10.1038/bjc.2012.296 (PMC3461151; doi:10.1038/bjc.2012.296)
Supplement: Supplementary Table S1 [file bjc2012296x2.doc]

|  | **ICCC** ^1^ | **No of cases** | **Age-sex-standardised rate per million** | | | | | | | | **AAPC (95% CI)** |
| --- | --- | --- | --- | --- | --- | --- | --- | --- | --- | --- | --- |
| **Diagnosis period** |  | **1966-2005** | **1966-1970** | **1971-1975** | **1976-1980** | **1981-1985** | **1986-1990** | **1991-1995** | **1996-2000** | **2001-2005** | **1966-2005** |
| **Age group (years)** |  |  |  |  |  |  |  |  |  |  |  |
| Under 1 | I-XII | 4818 | 137.9 | 133.3 | 143.6 | 157.6 | 164.8 | 177.7 | 192.3 | 222.6 | 1.4 (1.1 to 1.6) |
| 1-4 | I-XII | 20187 | 145.3 | 154.4 | 161.8 | 160.7 | 177.4 | 179.9 | 187.9 | 198.2 | 0.9 (0.7 to 1.0) |
| 5-9 | I-XII | 14777 | 81.3 | 87.9 | 94.0 | 89.6 | 98.2 | 105.4 | 107.8 | 113.0 | 0.9 (0.8 to 1.0) |
| 10-14 | I-XII | 14868 | 82.8 | 82.3 | 87.8 | 91.2 | 98.6 | 108.6 | 105.1 | 119.4 | 1.1 (1.0 to 1.2) |
|  |  |  |  |  |  |  |  |  |  |  |  |
| **Leukaemia** | **I** | **17850** | **34.0** | **37.5** | **38.4** | **37.4** | **40.1** | **40.9** | **43.0** | **45.8** | **0.7 (0.6 to 0.9)** |
| **CNS tumours** ^2^ | **III, Xa** | **13622** | **24.2** | **25.5** | **27.7** | **27.5** | **29.7** | **34.6** | **34.7** | **37.7** | **1.3 (1.2 to 1.5)** |
| **Non-CNS solid cancer** ^3^ | **Other** | **23178** | **44.4** | **43.8** | **47.3** | **48.7** | **54.1** | **55.7** | **56.2** | **61.6** | **1.0 (0.9 to 1.1)** |
|  |  |  |  |  |  |  |  |  |  |  |  |
| Lymphoma | II | 5579 | 10.8 | 10.5 | 11.9 | 11.4 | 12.1 | 12.7 | 13.4 | 14.3 | 0.8 (0.6 to 1.1) |
| Neuroblastoma | IV | 3552 | 7.9 | 7.2 | 6.6 | 7.4 | 9.0 | 7.8 | 8.7 | 9.4 | 0.6 (0.4 to 0.9) |
| Retinoblastoma | V | 1598 | 3.4 | 3.3 | 3.4 | 3.2 | 3.6 | 4.3 | 3.6 | 4.1 | 0.6 (0.2 to 1.1) |
| Renal cancer | VI | 3214 | 6.5 | 6.7 | 7.1 | 6.6 | 7.2 | 7.2 | 7.7 | 8.8 | 0.7 (0.4 to 1.0) |
| Hepatic cancer | VII | 483 | 0.7 | 0.7 | 0.9 | 1.0 | 1.2 | 1.2 | 1.4 | 1.8 | 2.5 (1.7 to 3.3) |
| Bone cancer | VIII | 2427 | 4.3 | 4.9 | 5.5 | 5.6 | 5.7 | 5.4 | 5.1 | 5.8 | 0.5 (0.2 to 0.9) |
| Soft-tissue sarcoma | IX | 3549 | 5.6 | 6.4 | 6.7 | 7.4 | 8.6 | 9.9 | 9.0 | 9.7 | 1.6 (1.3 to 1.9) |
| Germ-cell/gonadal ^4^ | Xb-Xe | 1224 | 2.3 | 2.1 | 2.4 | 2.9 | 3.2 | 2.7 | 3.3 | 3.2 | 1.1 (0.6 to 1.6) |
| Melanoma/carcinoma ^5^ | XI | 1255 | 2.2 | 1.8 | 2.4 | 2.7 | 3.0 | 3.3 | 3.4 | 3.6 | 1.8 (1.3 to 2.3) |
| Other/unspecified ^6^ | XII | 297 | 0.8 | 0.3 | 0.3 | 0.5 | 0.6 | 1.2 | 0.7 | 1.0 | 2.2 (1.2 to 3.2) |
|  |  |  |  |  |  |  |  |  |  |  |  |
| **Total childhood cancer** | **I-XII** | **54650** | **102.6** | **106.8** | **113.3** | **113.6** | **123.9** | **131.2** | **133.9** | **145.1** | **1.0 (0.9 to 1.1)** |
|  |  |  |  |  |  |  |  |  |  |  |  |

**Table S1. Childhood cancer, Great Britain 1966-2005.** Number of recorded cases. Age-sex-standardised rates per million by 5-year period of diagnosis. Annual average percent change (AAPC) with estimated 95% confidence interval (CI).

^1^ International Classification of Childhood Cancer, third edition, excluding skin carcinoma and non-CNS neoplasms treated as uncertain/benign in earlier coding systems. **^2^** Intracranial/intraspinal tumours. ^3^ All cancer except leukaemia and CNS tumours. ^4^ Non-CNS germ-cell and gonadal cancer. ^5^ Malignant melanoma, and carcinoma of miscellaneous sites not otherwise specified.  ^6^ Other and unspecified malignant neoplasms.
